# Multiple crusted plaques on lower portion of the legs and abdomen

**DOI:** 10.1016/j.jdcr.2023.07.043

**Published:** 2023-11-08

**Authors:** Uma Desai, Anand Rajpara, Dominic J. Wu

**Affiliations:** a University of Kansas School of Medicine, Kansas City, Missouri; b Department of Dermatology, University of Missouri–Kansas City, Kansas City, Missouri

**Keywords:** infection, papules, plaques, rickettsialpox, rodent

A 33-year-old woman with obstructive sleep apnea, depression, alcohol use disorder, and thiamine deficiency presented with fever and numerous erythematous crusted papules and plaques with central necrotic-appearing eschar on her lower portion of the legs and abdomen (Figure 1). The lower portion of the leg lesions began 2 weeks before admission as vesicles, which developed into crusted erythematous plaques with central necrotic eschar. She was found on the floor of her mobile home, surrounded by scattered rodent feces. The patient did not present with any additional systemic symptoms apart from what is listed. Laboratory findings were significant for thrombocytopenia and mild leukopenia. A 1,3-β-D-glucan (Fungitell) assay was negative. Histopathology was nonspecific and revealed eschar with mixed inflammation and vascular congestion with negative fungal and bacterial pathogens (Figure 2).

**Fig 1:**
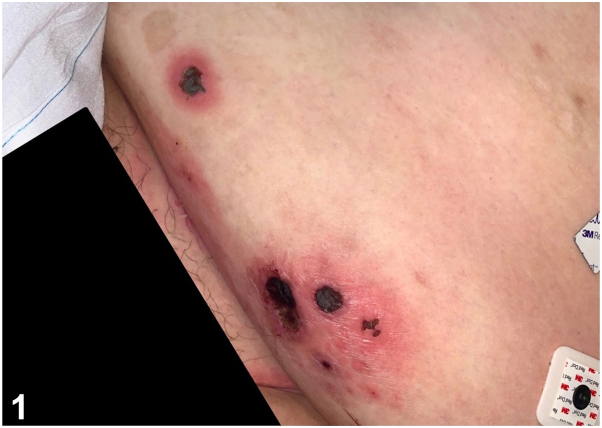
▪▪▪

**Fig 2:**
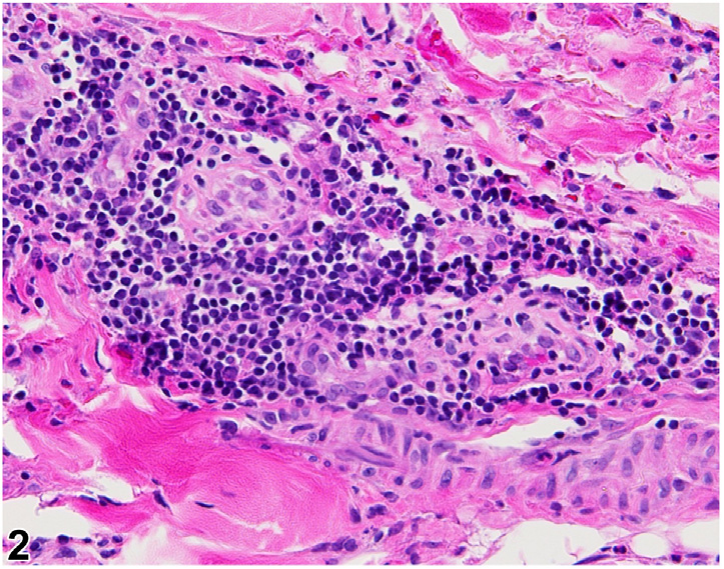
▪▪▪

**Question 1: What is the patient’s most likely diagnosis?**

A. Ecthyma gangrenosum
B. Septic vasculitis
C. Varicella
D. Rickettsialpox
E. Tularemia

**Answers:**

**A.** Ecthyma gangrenosum – Incorrect. Ecthyma gangrenosum is most frequently caused by *Pseudomonas aeruginosa* and presents as erythematous ulcerated hemorrhagic papules and plaques with central necrotic eschar. It is usually also associated with immunosuppression. Histopathology would reveal numerous gram-negative rods (*P aeruginosa*).
**B.** Septic vasculitis^1^ – Incorrect. Septic vasculitis is a mixed vessel vasculitis which may occur secondary to a severe systemic infection. Vasculitis would be present on histopathology and, clinically, lesions would be expected to be more widespread with palpable purpura and hemorrhagic bullae.
**C.** Varicella – Incorrect. A primary varicella-zoster virus infection results in varicella or herpes zoster manifesting as vesicles on an erythematous base either in generalized or dermatomal distribution, which may resemble rickettsialpox. However, varicella lesions tend to lack the central black necrotic eschar this patient’s lesions exhibited. Upon skin biopsy, one would expect to see numerous multinucleated keratinocytes.
**D.** Rickettsialpox – Correct. The presence of rodent feces surrounding the patient and history of poor hygiene was key to diagnosing this patient with rickettsialpox. Other diagnostic clues included the presence of the classic central necrotic eschar with a surrounding erythematous halo (Figure 3). Immunohistochemical staining, polymerase chain reaction, or culture specific to *Rickettsia akari* may be more sensitive. Rickettsialpox is caused by *R akari*, a gram-negative bacterium that is typically transmitted to humans by painless mouse mite (*Liponyssoides saguineus*) bites. Skin biopsy results tend to be nonspecific, and bacterial organisms may not be readily visible.

**Fig 3:**
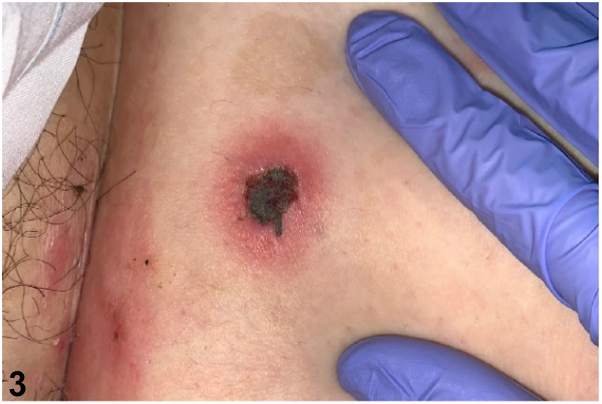
▪▪▪
**E.** Tularemia^2^ – Incorrect. Tularemia is caused by *Francisella tularensis*, which is typically acquired by humans after direct or indirect contact with bodily fluids of animal carriers, such as rabbits. Although the skin lesions of tularemia resemble that of rickettsialpox, they tend to be more localized to the point of inoculation. Furthermore, patients typically present with systemic symptoms including lymphadenopathy, pneumonia, sore throat, and myalgia, which were absent in this patient.

**Question 2: What is the treatment of choice for this patient?**

A. Doxycycline
B. Cephalexin
C. Prednisone
D. Chloramphenicol
E. Streptomycin

**Answers:**

**A.** Doxycycline – Correct. Rickettsialpox tends to be mild and self-limiting; however, doxycycline may decrease the recovery time to a few days and is considered the treatment of choice, even for children and pregnant patients. This patient’s condition resolved after taking doxycycline 100 mg twice daily for 10 days.
**B.** Cephalexin – Incorrect. First-generation cephalosporins work well against gram-positive bacteria; however, *R akari* is a gram-negative bacterium and this is not the preferred treatment.
**C.** Prednisone – Incorrect. Prednisone alone would likely not benefit the patient in her recovery. Antibiotics such as doxycycline is the first-line treatment for rickettsialpox.
**D.** Chloramphenicol – Incorrect. Although chloramphenicol is an acceptable alternative antibiotic for rickettsialpox, it is not typically used due to its significant side effect profile.
**E.** Streptomycin – Incorrect. This medication is typically associated with treatment of pulmonary tuberculosis, a moderate-to-severe infection. Rickettsialpox is a milder infection.

**Question 3: What is the key diagnostic indicator in this patient’s history and laboratory workup?**

A. Human feces
B. Rodent feces
C. Mild leukopenia
D. History of alcohol use disorder
E. Thiamine deficiency

**Answers:**

**A.** Human feces – Incorrect. Human feces may cause diseases such as cryptosporidiosis, typhoid, cholera, and hepatitis. However, it is unlikely that an infection from human feces will cause rickettsialpox.
**B.** Rodent feces – Correct. Rickettsialpox is caused by *R akari*, a gram-negative bacterium typically transmitted to humans by painless mouse mite (*L saguineus*) bites.
**C.** Mild leukopenia – Incorrect. General laboratory workup for rickettsialpox is typically nonspecific and may or may not reveal leukopenia.
**D.** History of alcohol use disorder – Incorrect. Although alcohol use disorder may worsen skin conditions such as psoriasis and eczema, it is not a cause of rickettsialpox.
**E.** Thiamine deficiency – Incorrect. Thiamine deficiency is associated with alcohol use disorder and causes beriberi. It can worsen pellagra symptoms; however, it likely did not cause her infection.

## Conclusion

Rickettsialpox is a disease caused by *R akari* and is typically self-limiting. The bacterium is transmitted via a mite bite, which creates central eschar at the site of infection as well as a papular or vesicular rash.

## Conflicts of interest

None disclosed.
